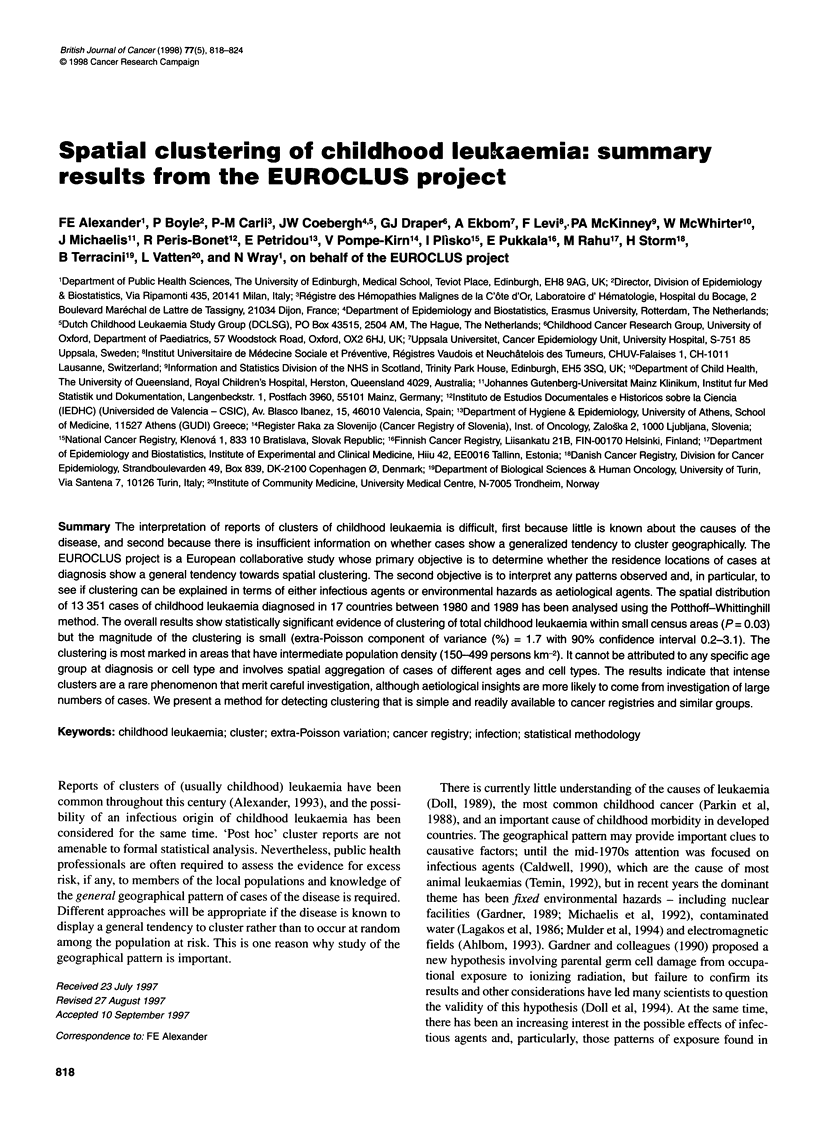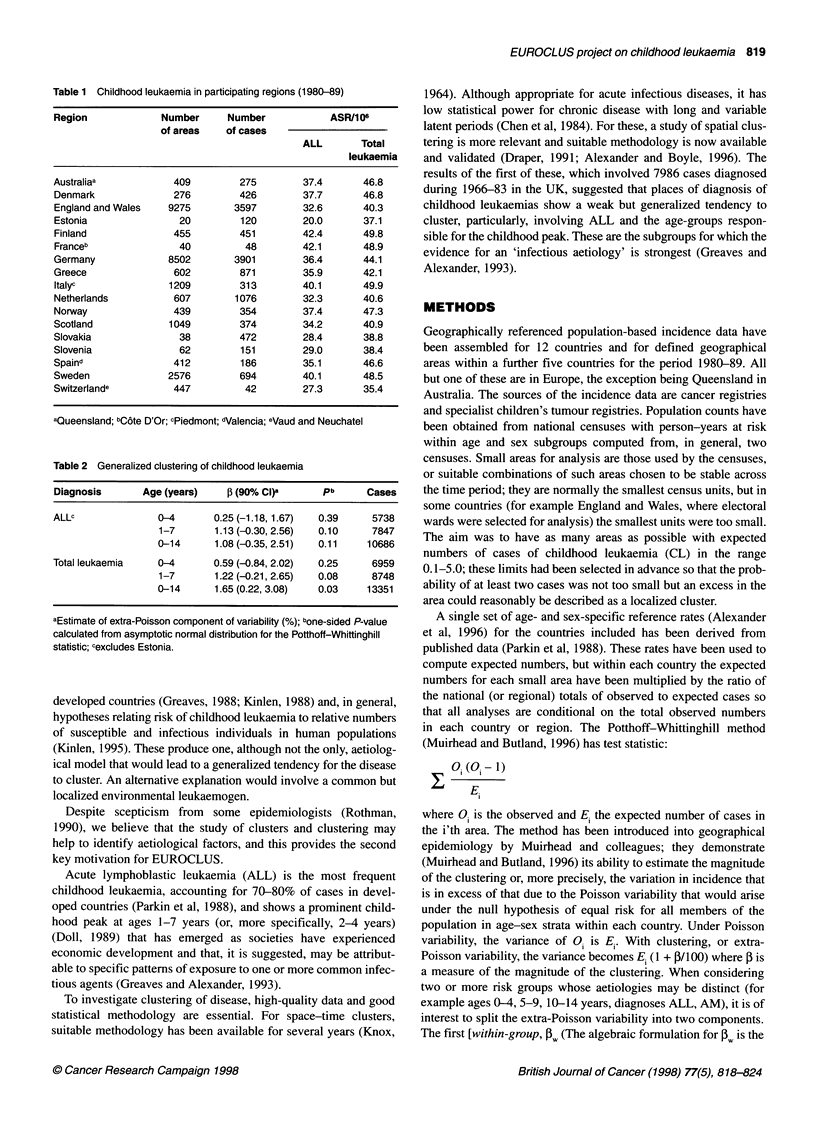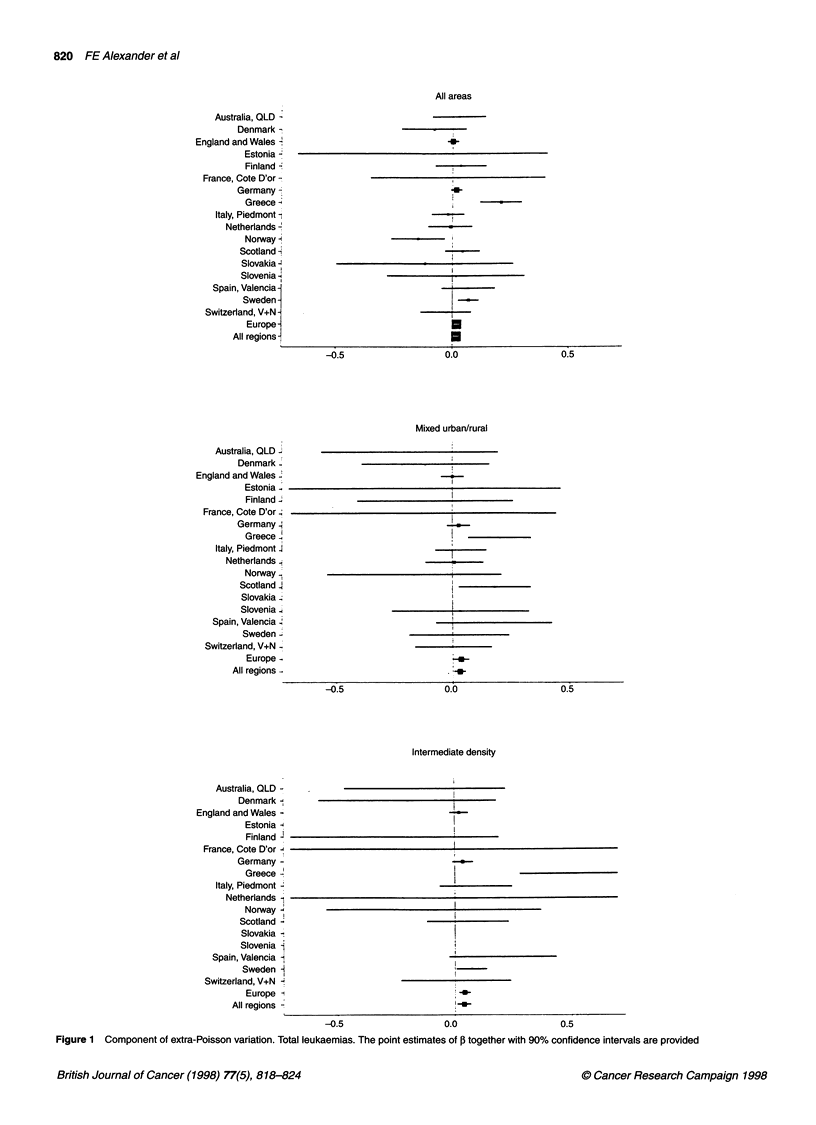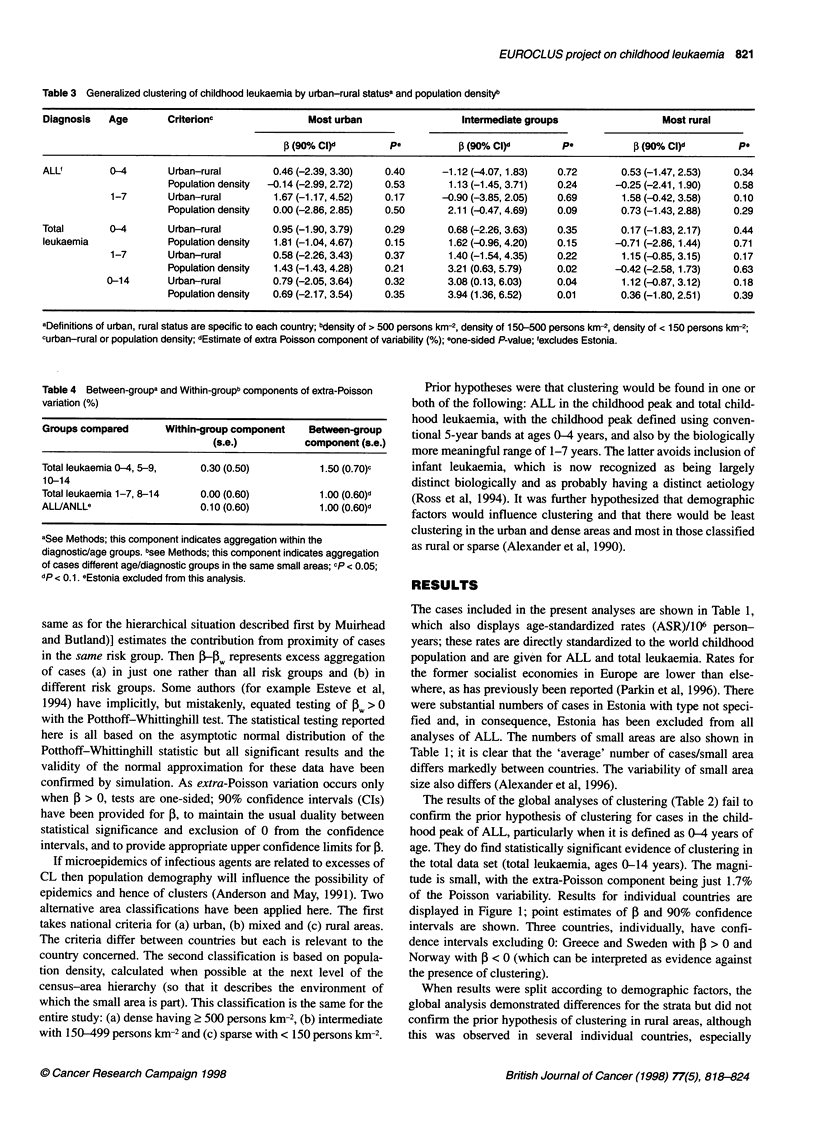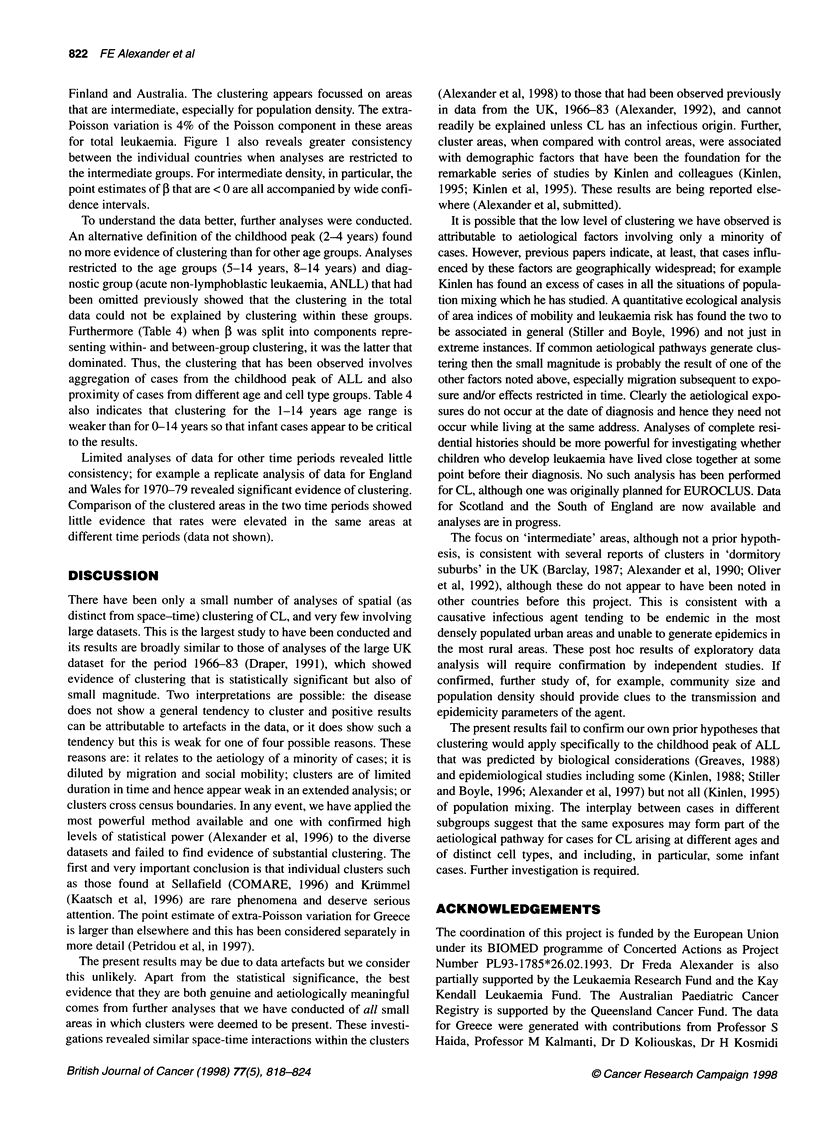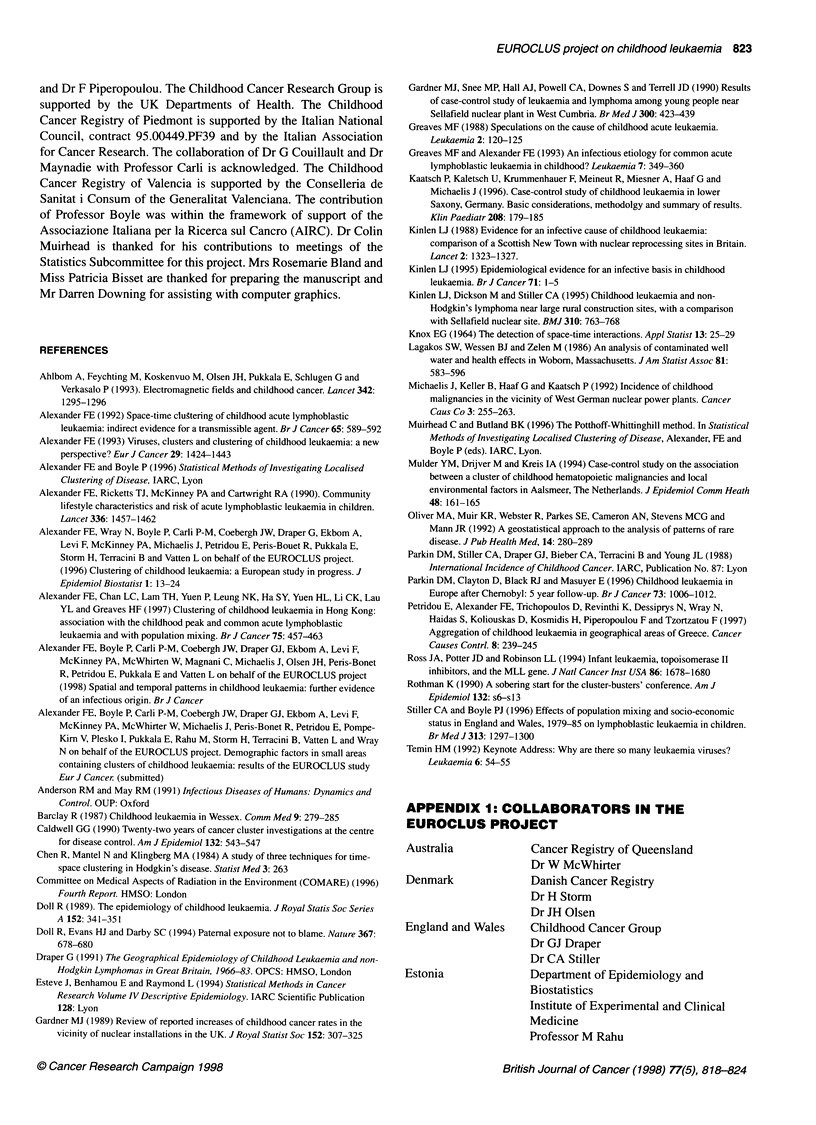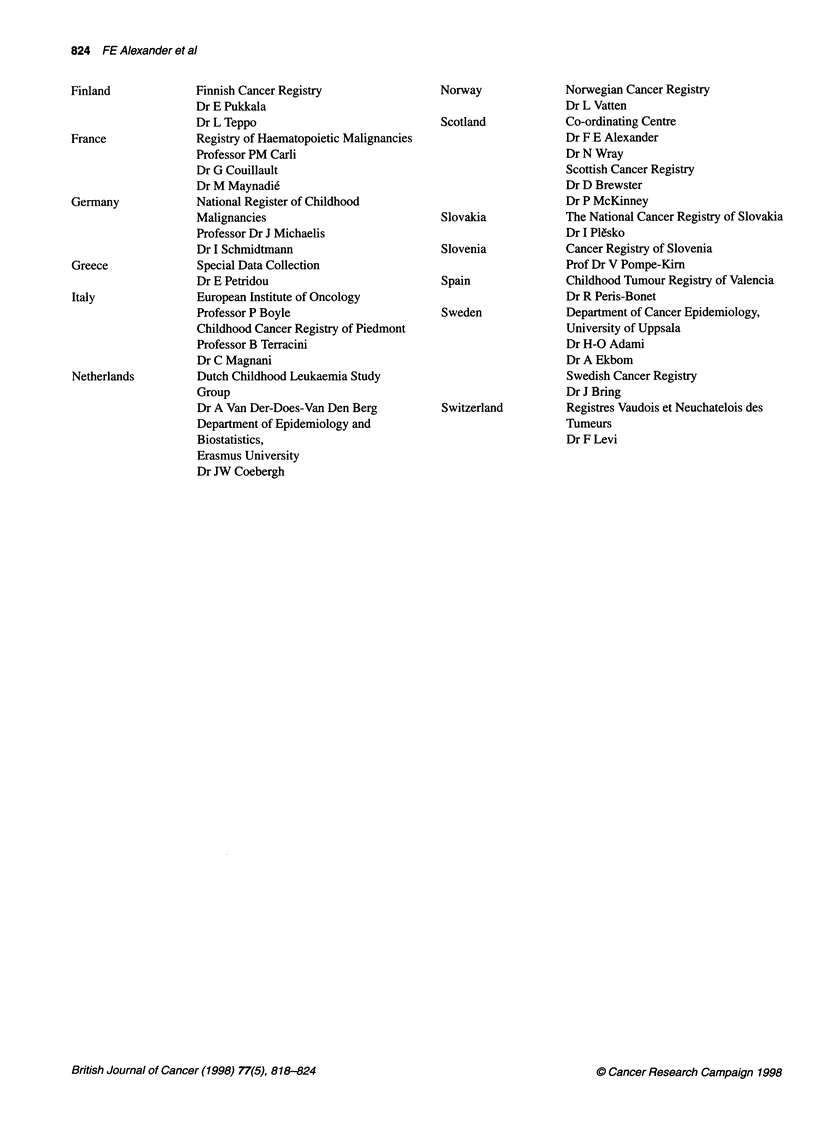# Spatial clustering of childhood leukaemia: summary results from the EUROCLUS project.

**DOI:** 10.1038/bjc.1998.133

**Published:** 1998-03

**Authors:** F. E. Alexander, P. Boyle, P. M. Carli, J. W. Coebergh, G. J. Draper, A. Ekbom, F. Levi, P. A. McKinney, W. McWhirter, J. Michaelis, R. Peris-Bonet, E. Petridou, V. Pompe-Kirn, I. PlÃ¬sko, E. Pukkala, M. Rahu, H. Storm, B. Terracini, L. Vatten, N. Wray

**Affiliations:** Department of Public Health Sciences, The University of Edinburgh, Medical School, UK.

## Abstract

The interpretation of reports of clusters of childhood leukaemia is difficult, first because little is known about the causes of the disease, and second because there is insufficient information on whether cases show a generalized tendency to cluster geographically. The EUROCLUS project is a European collaborative study whose primary objective is to determine whether the residence locations of cases at diagnosis show a general tendency towards spatial clustering. The second objective is to interpret any patterns observed and, in particular, to see if clustering can be explained in terms of either infectious agents or environmental hazards as aetiological agents. The spatial distribution of 13351 cases of childhood leukaemia diagnosed in 17 countries between 1980 and 1989 has been analysed using the Potthoff-Whittinghill method. The overall results show statistically significant evidence of clustering of total childhood leukaemia within small census areas (P=0.03) but the magnitude of the clustering is small (extra-Poisson component of variance (%) = 1.7 with 90% confidence interval 0.2-3.1). The clustering is most marked in areas that have intermediate population density (150-499 persons km[-2]). It cannot be attributed to any specific age group at diagnosis or cell type and involves spatial aggregation of cases of different ages and cell types. The results indicate that intense clusters are a rare phenomenon that merit careful investigation, although aetiological insights are more likely to come from investigation of large numbers of cases. We present a method for detecting clustering that is simple and readily available to cancer registries and similar groups.


British Journal of Cancer (1998) 77(5), 818–824
© 1998 Cancer Research Campaign

### Spatial clustering of childhood leukaemia: summary results from the EUROCLUS project

**FE Alexander[1], P Boyle[2], P-M Carli[3], JW Coebergh[4,5], GJ Draper[6], A Ekbom[7], F Levi[8], PA McKinney[9], W McWhirter[10], J Michaelis[11], R Peris-Bonet[12], E Petridou[13], V Pompe-Kirn[14], I Plìsko[15], E Pukkala[16], M Rahu[17], H Storm[18], B Terracini[19], L Vatten[20], and N Wray[1], on behalf of the EUROCLUS project**

[1]Department of Public Health Sciences, The University of Edinburgh, Medical School, Teviot Place, Edinburgh, EH8 9AG, UK; [2]Director, Division of Epidemiology & Biostatistics, Via Ripamonti 435, 20141 Milan, Italy; [3]Régistre des Hémopathies Malignes de la C'ôte d'Or, Laboratoire d' Hématologie, Hospital du Bocage, 2 Boulevard Maréchal de Lattre de Tassigny, 21034 Dijon, France; [4]Department of Epidemiology and Biostatistics, Erasmus University, Rotterdam, The Netherlands; [5]Dutch Childhood Leukaemia Study Group (DCLSG), PO Box 43515, 2504 AM, The Hague, The Netherlands; [6]Childhood Cancer Research Group, University of Oxford, Department of Paediatrics, 57 Woodstock Road, Oxford, OX2 6HJ, UK; [7]Uppsala Universitet, Cancer Epidemiology Unit, University Hospital, S-751 85 Uppsala, Sweden; [8]Institut Universitaire de Médecine Sociale et Préventive, Régistres Vaudois et Neuchâtelois des Tumeurs, CHUV-Falaises 1, CH-1011 Lausanne, Switzerland; [9]Information and Statistics Division of the NHS in Scotland, Trinity Park House, Edinburgh, EH5 3SQ, UK; [10]Department of Child Health, The University of Queensland, Royal Children's Hospital, Herston, Queensland 4029, Australia; [11]Johannes Gutenberg-Universitat Mainz Klinikum, Institut fur Med Statistik und Dokumentation, Langenbeckstr. 1, Postfach 3960, 55101 Mainz, Germany; [12]Instituto de Estudios Documentales e Historicos sobre la Ciencia (IEDHC) (Universided de Valencia – CSIC), Av. Blasco Ibanez, 15, 46010 Valencia, Spain; [13]Department of Hygiene & Epidemiology, University of Athens, School of Medicine, 11527 Athens (GUDI) Greece; [14]Register Raka za Slovenijo (Cancer Registry of Slovenia), Inst. of Oncology, Zaloška 2, 1000 Ljubljana, Slovenia; [15]National Cancer Registry, Klenová 1, 833 10 Bratislava, Slovak Republic; [16]Finnish Cancer Registry, Liisankatu 21B, FIN-00170 Helsinki, Finland; [17]Department of Epidemiology and Biostatistics, Institute of Experimental and Clinical Medicine, Hiiu 42, EE0016 Tallinn, Estonia; [18]Danish Cancer Registry, Division for Cancer Epidemiology, Strandboulevarden 49, Box 839, DK-2100 Copenhagen Ø, Denmark; [19]Department of Biological Sciences & Human Oncology, University of Turin, Via Santena 7, 10126 Turin, Italy; [20]Institute of Community Medicine, University Medical Centre, N-7005 Trondheim, Norway

**Summary** The interpretation of reports of clusters of childhood leukaemia is difficult, first because little is known about the causes of the disease, and second because there is insufficient information on whether cases show a generalized tendency to cluster geographically. The EUROCLUS project is a European collaborative study whose primary objective is to determine whether the residence locations of cases at diagnosis show a general tendency towards spatial clustering. The second objective is to interpret any patterns observed and, in particular, to see if clustering can be explained in terms of either infectious agents or environmental hazards as aetiological agents. The spatial distribution of 13 351 cases of childhood leukaemia diagnosed in 17 countries between 1980 and 1989 has been analysed using the Potthoff–Whittinghill method. The overall results show statistically significant evidence of clustering of total childhood leukaemia within small census areas ($P = 0.03$) but the magnitude of the clustering is small (extra-Poisson component of variance (%) = 1.7 with 90% confidence interval 0.2–3.1). The clustering is most marked in areas that have intermediate population density (150–499 persons km$^{-2}$). It cannot be attributed to any specific age group at diagnosis or cell type and involves spatial aggregation of cases of different ages and cell types. The results indicate that intense clusters are a rare phenomenon that merit careful investigation, although aetiological insights are more likely to come from investigation of large numbers of cases. We present a method for detecting clustering that is simple and readily available to cancer registries and similar groups.

**Keywords:** childhood leukaemia; cluster; extra-Poisson variation; cancer registry; infection; statistical methodology

Reports of clusters of (usually childhood) leukaemia have been common throughout this century (Alexander, 1993), and the possibility of an infectious origin of childhood leukaemia has been considered for the same time. 'Post hoc' cluster reports are not amenable to formal statistical analysis. Nevertheless, public health professionals are often required to assess the evidence for excess risk, if any, to members of the local populations and knowledge of the *general* geographical pattern of cases of the disease is required. Different approaches will be appropriate if the disease is known to display a general tendency to cluster rather than to occur at random among the population at risk. This is one reason why study of the geographical pattern is important.

*Received 23 July 1997*
*Revised 27 August 1997*
*Accepted 10 September 1997*

*Correspondence to:* FE Alexander

There is currently little understanding of the causes of leukaemia (Doll, 1989), the most common childhood cancer (Parkin et al, 1988), and an important cause of childhood morbidity in developed countries. The geographical pattern may provide important clues to causative factors; until the mid-1970s attention was focused on infectious agents (Caldwell, 1990), which are the cause of most animal leukaemias (Temin, 1992), but in recent years the dominant theme has been *fixed* environmental hazards – including nuclear facilities (Gardner, 1989; Michaelis et al, 1992), contaminated water (Lagakos et al, 1986; Mulder et al, 1994) and electromagnetic fields (Ahlbom, 1993). Gardner and colleagues (1990) proposed a new hypothesis involving parental germ cell damage from occupational exposure to ionizing radiation, but failure to confirm its results and other considerations have led many scientists to question the validity of this hypothesis (Doll et al, 1994). At the same time, there has been an increasing interest in the possible effects of infectious agents and, particularly, those patterns of exposure found in

**Table 1** Childhood leukaemia in participating regions (1980–89)

| Region | Number of areas | Number of cases | ASR/$10^6$ | |
|---|---|---|---|---|
| | | | ALL | Total leukaemia |
| Australia[a] | 409 | 275 | 37.4 | 46.8 |
| Denmark | 276 | 426 | 37.7 | 46.8 |
| England and Wales | 9275 | 3597 | 32.6 | 40.3 |
| Estonia | 20 | 120 | 20.0 | 37.1 |
| Finland | 455 | 451 | 42.4 | 49.8 |
| France[b] | 40 | 48 | 42.1 | 48.9 |
| Germany | 8502 | 3901 | 36.4 | 44.1 |
| Greece | 602 | 871 | 35.9 | 42.1 |
| Italy[c] | 1209 | 313 | 40.1 | 49.9 |
| Netherlands | 607 | 1076 | 32.3 | 40.6 |
| Norway | 439 | 354 | 37.4 | 47.3 |
| Scotland | 1049 | 374 | 34.2 | 40.9 |
| Slovakia | 38 | 472 | 28.4 | 38.8 |
| Slovenia | 62 | 151 | 29.0 | 38.4 |
| Spain[d] | 412 | 186 | 35.1 | 46.6 |
| Sweden | 2576 | 694 | 40.1 | 48.5 |
| Switzerland[e] | 447 | 42 | 27.3 | 35.4 |

[a]Queensland; [b]Côte D'Or; [c]Piedmont; [d]Valencia; [e]Vaud and Neuchatel

**Table 2** Generalized clustering of childhood leukaemia

| Diagnosis | Age (years) | β (90% CI)[a] | $P^b$ | Cases |
|---|---|---|---|---|
| ALL[c] | 0–4 | 0.25 (–1.18, 1.67) | 0.39 | 5738 |
| | 1–7 | 1.13 (–0.30, 2.56) | 0.10 | 7847 |
| | 0–14 | 1.08 (–0.35, 2.51) | 0.11 | 10686 |
| Total leukaemia | 0–4 | 0.59 (–0.84, 2.02) | 0.25 | 6959 |
| | 1–7 | 1.22 (–0.21, 2.65) | 0.08 | 8748 |
| | 0–14 | 1.65 (0.22, 3.08) | 0.03 | 13351 |

[a]Estimate of extra-Poisson component of variability (%); [b]one-sided *P*-value calculated from asymptotic normal distribution for the Potthoff–Whittinghill statistic; [c]excludes Estonia.

developed countries (Greaves, 1988; Kinlen, 1988) and, in general, hypotheses relating risk of childhood leukaemia to relative numbers of susceptible and infectious individuals in human populations (Kinlen, 1995). These produce one, although not the only, aetiological model that would lead to a generalized tendency for the disease to cluster. An alternative explanation would involve a common but localized environmental leukaemogen.

Despite scepticism from some epidemiologists (Rothman, 1990), we believe that the study of clusters and clustering may help to identify aetiological factors, and this provides the second key motivation for EUROCLUS.

Acute lymphoblastic leukaemia (ALL) is the most frequent childhood leukaemia, accounting for 70–80% of cases in developed countries (Parkin et al, 1988), and shows a prominent childhood peak at ages 1–7 years (or, more specifically, 2–4 years) (Doll, 1989) that has emerged as societies have experienced economic development and that, it is suggested, may be attributable to specific patterns of exposure to one or more common infectious agents (Greaves and Alexander, 1993).

To investigate clustering of disease, high-quality data and good statistical methodology are essential. For space–time clusters, suitable methodology has been available for several years (Knox, 1964). Although appropriate for acute infectious diseases, it has low statistical power for chronic disease with long and variable latent periods (Chen et al, 1984). For these, a study of spatial clustering is more relevant and suitable methodology is now available and validated (Draper, 1991; Alexander and Boyle, 1996). The results of the first of these, which involved 7986 cases diagnosed during 1966–83 in the UK, suggested that places of diagnosis of childhood leukaemias show a weak but generalized tendency to cluster, particularly, involving ALL and the age-groups responsible for the childhood peak. These are the subgroups for which the evidence for an 'infectious aetiology' is strongest (Greaves and Alexander, 1993).

### METHODS

Geographically referenced population-based incidence data have been assembled for 12 countries and for defined geographical areas within a further five countries for the period 1980–89. All but one of these are in Europe, the exception being Queensland in Australia. The sources of the incidence data are cancer registries and specialist children's tumour registries. Population counts have been obtained from national censuses with person–years at risk within age and sex subgroups computed from, in general, two censuses. Small areas for analysis are those used by the censuses, or suitable combinations of such areas chosen to be stable across the time period; they are normally the smallest census units, but in some countries (for example England and Wales, where electoral wards were selected for analysis) the smallest units were too small. The aim was to have as many areas as possible with expected numbers of cases of childhood leukaemia (CL) in the range 0.1–5.0; these limits had been selected in advance so that the probability of at least two cases was not too small but an excess in the area could reasonably be described as a localized cluster.

A single set of age- and sex-specific reference rates (Alexander et al, 1996) for the countries included has been derived from published data (Parkin et al, 1988). These rates have been used to compute expected numbers, but within each country the expected numbers for each small area have been multiplied by the ratio of the national (or regional) totals of observed to expected cases so that all analyses are conditional on the total observed numbers in each country or region. The Potthoff–Whittinghill method (Muirhead and Butland, 1996) has test statistic:

$$\sum \frac{O_i(O_i - 1)}{E_i}$$

where $O_i$ is the observed and $E_i$ the expected number of cases in the i'th area. The method has been introduced into geographical epidemiology by Muirhead and colleagues; they demonstrate (Muirhead and Butland, 1996) its ability to estimate the magnitude of the clustering or, more precisely, the variation in incidence that is in excess of that due to the Poisson variability that would arise under the null hypothesis of equal risk for all members of the population in age–sex strata within each country. Under Poisson variability, the variance of $O_i$ is $E_i$. With clustering, or extra-Poisson variability, the variance becomes $E_i\,(1 + \beta/100)$ where β is a measure of the magnitude of the clustering. When considering two or more risk groups whose aetiologies may be distinct (for example ages 0–4, 5–9, 10–14 years, diagnoses ALL, AM), it is of interest to split the extra-Poisson variability into two components. The first [*within-group*, $\beta_w$ (The algebraic formulation for $\beta_w$ is the

**Figure 1** Component of extra-Poisson variation. Total leukaemias. The point estimates of β together with 90% confidence intervals are provided

*© Cancer Research Campaign 1998*

**Table 3** Generalized clustering of childhood leukaemia by urban–rural status[a] and population density[b]

| Diagnosis | Age | Criterion[c] | Most urban | | Intermediate groups | | Most rural | |
|---|---|---|---|---|---|---|---|---|
| | | | β (90% CI)[d] | *P*[e] | β (90% CI)[d] | *P*[e] | β (90% CI)[d] | *P*[e] |
| ALL[f] | 0–4 | Urban–rural | 0.46 (−2.39, 3.30) | 0.40 | −1.12 (−4.07, 1.83) | 0.72 | 0.53 (−1.47, 2.53) | 0.34 |
| | | Population density | −0.14 (−2.99, 2.72) | 0.53 | 1.13 (−1.45, 3.71) | 0.24 | −0.25 (−2.41, 1.90) | 0.58 |
| | 1–7 | Urban–rural | 1.67 (−1.17, 4.52) | 0.17 | −0.90 (−3.85, 2.05) | 0.69 | 1.58 (−0.42, 3.58) | 0.10 |
| | | Population density | 0.00 (−2.86, 2.85) | 0.50 | 2.11 (−0.47, 4.69) | 0.09 | 0.73 (−1.43, 2.88) | 0.29 |
| Total leukaemia | 0–4 | Urban–rural | 0.95 (−1.90, 3.79) | 0.29 | 0.68 (−2.26, 3.63) | 0.35 | 0.17 (−1.83, 2.17) | 0.44 |
| | | Population density | 1.81 (−1.04, 4.67) | 0.15 | 1.62 (−0.96, 4.20) | 0.15 | −0.71 (−2.86, 1.44) | 0.71 |
| | 1–7 | Urban–rural | 0.58 (−2.26, 3.43) | 0.37 | 1.40 (−1.54, 4.35) | 0.22 | 1.15 (−0.85, 3.15) | 0.17 |
| | | Population density | 1.43 (−1.43, 4.28) | 0.21 | 3.21 (0.63, 5.79) | 0.02 | −0.42 (−2.58, 1.73) | 0.63 |
| | 0–14 | Urban–rural | 0.79 (−2.05, 3.64) | 0.32 | 3.08 (0.13, 6.03) | 0.04 | 1.12 (−0.87, 3.12) | 0.18 |
| | | Population density | 0.69 (−2.17, 3.54) | 0.35 | 3.94 (1.36, 6.52) | 0.01 | 0.36 (−1.80, 2.51) | 0.39 |

[a]Definitions of urban, rural status are specific to each country; [b]density of > 500 persons $km^{-2}$, density of 150–500 persons $km^{-2}$, density of < 150 persons $km^{-2}$; [c]urban–rural or population density; [d]Estimate of extra Poisson component of variability (%); [e]one-sided *P*-value; [f]excludes Estonia.

**Table 4** Between-group[a] and Within-group[b] components of extra-Poisson variation (%)

| Groups compared | Within-group component (s.e.) | Between-group component (s.e.) |
|---|---|---|
| Total leukaemia 0–4, 5–9, 10–14 | 0.30 (0.50) | 1.50 (0.70)[c] |
| Total leukaemia 1–7, 8–14 | 0.00 (0.60) | 1.00 (0.60)[d] |
| ALL/ANLL[e] | 0.10 (0.60) | 1.00 (0.60)[d] |

[a]See Methods; this component indicates aggregation within the diagnostic/age groups. [b]see Methods; this component indicates aggregation of cases different age/diagnostic groups in the same small areas; [c]$P < 0.05$; [d]$P < 0.1$. [e]Estonia excluded from this analysis.

same as for the hierarchical situation described first by Muirhead and Butland)] estimates the contribution from proximity of cases in the *same* risk group. Then $\beta - \beta_w$ represents excess aggregation of cases (a) in just one rather than all risk groups and (b) in different risk groups. Some authors (for example Esteve et al, 1994) have implicitly, but mistakenly, equated testing of $\beta_w > 0$ with the Potthoff–Whittinghill test. The statistical testing reported here is all based on the asymptotic normal distribution of the Potthoff–Whittinghill statistic but all significant results and the validity of the normal approximation for these data have been confirmed by simulation. As *extra*-Poisson variation occurs only when $\beta > 0$, tests are one-sided; 90% confidence intervals (CIs) have been provided for β, to maintain the usual duality between statistical significance and exclusion of 0 from the confidence intervals, and to provide appropriate upper confidence limits for β.

If microepidemics of infectious agents are related to excesses of CL then population demography will influence the possibility of epidemics and hence of clusters (Anderson and May, 1991). Two alternative area classifications have been applied here. The first takes national criteria for (a) urban, (b) mixed and (c) rural areas. The criteria differ between countries but each is relevant to the country concerned. The second classification is based on population density, calculated when possible at the next level of the census–area hierarchy (so that it describes the environment of which the small area is part). This classification is the same for the entire study: (a) dense having ≥ 500 persons $km^{-2}$, (b) intermediate with 150–499 persons $km^{-2}$ and (c) sparse with < 150 persons $km^{-2}$.

Prior hypotheses were that clustering would be found in one or both of the following: ALL in the childhood peak and total childhood leukaemia, with the childhood peak defined using conventional 5-year bands at ages 0–4 years, and also by the biologically more meaningful range of 1–7 years. The latter avoids inclusion of infant leukaemia, which is now recognized as being largely distinct biologically and as probably having a distinct aetiology (Ross et al, 1994). It was further hypothesized that demographic factors would influence clustering and that there would be least clustering in the urban and dense areas and most in those classified as rural or sparse (Alexander et al, 1990).

### RESULTS

The cases included in the present analyses are shown in Table 1, which also displays age-standardized rates (ASR)/$10^6$ person–years; these rates are directly standardized to the world childhood population and are given for ALL and total leukaemia. Rates for the former socialist economies in Europe are lower than elsewhere, as has previously been reported (Parkin et al, 1996). There were substantial numbers of cases in Estonia with type not specified and, in consequence, Estonia has been excluded from all analyses of ALL. The numbers of small areas are also shown in Table 1; it is clear that the 'average' number of cases/small area differs markedly between countries. The variability of small area size also differs (Alexander et al, 1996).

The results of the global analyses of clustering (Table 2) fail to confirm the prior hypothesis of clustering for cases in the childhood peak of ALL, particularly when it is defined as 0–4 years of age. They do find statistically significant evidence of clustering in the total data set (total leukaemia, ages 0–14 years). The magnitude is small, with the extra-Poisson component being just 1.7% of the Poisson variability. Results for individual countries are displayed in Figure 1; point estimates of β and 90% confidence intervals are shown. Three countries, individually, have confidence intervals excluding 0: Greece and Sweden with $\beta > 0$ and Norway with $\beta < 0$ (which can be interpreted as evidence against the presence of clustering).

When results were split according to demographic factors, the global analysis demonstrated differences for the strata but did not confirm the prior hypothesis of clustering in rural areas, although this was observed in several individual countries, especially

*© Cancer Research Campaign 1998* 

Finland and Australia. The clustering appears focussed on areas that are intermediate, especially for population density. The extra-Poisson variation is 4% of the Poisson component in these areas for total leukaemia. Figure 1 also reveals greater consistency between the individual countries when analyses are restricted to the intermediate groups. For intermediate density, in particular, the point estimates of $\beta$ that are < 0 are all accompanied by wide confidence intervals.

To understand the data better, further analyses were conducted. An alternative definition of the childhood peak (2–4 years) found no more evidence of clustering than for other age groups. Analyses restricted to the age groups (5–14 years, 8–14 years) and diagnostic group (acute non-lymphoblastic leukaemia, ANLL) that had been omitted previously showed that the clustering in the total data could not be explained by clustering within these groups. Furthermore (Table 4) when $\beta$ was split into components representing within- and between-group clustering, it was the latter that dominated. Thus, the clustering that has been observed involves aggregation of cases from the childhood peak of ALL and also proximity of cases from different age and cell type groups. Table 4 also indicates that clustering for the 1–14 years age range is weaker than for 0–14 years so that infant cases appear to be critical to the results.

Limited analyses of data for other time periods revealed little consistency; for example a replicate analysis of data for England and Wales for 1970–79 revealed significant evidence of clustering. Comparison of the clustered areas in the two time periods showed little evidence that rates were elevated in the same areas at different time periods (data not shown).

### DISCUSSION

There have been only a small number of analyses of spatial (as distinct from space–time) clustering of CL, and very few involving large datasets. This is the largest study to have been conducted and its results are broadly similar to those of analyses of the large UK dataset for the period 1966–83 (Draper, 1991), which showed evidence of clustering that is statistically significant but also of small magnitude. Two interpretations are possible: the disease does not show a general tendency to cluster and positive results can be attributable to artefacts in the data, or it does show such a tendency but this is weak for one of four possible reasons. These reasons are: it relates to the aetiology of a minority of cases; it is diluted by migration and social mobility; clusters are of limited duration in time and hence appear weak in an extended analysis; or clusters cross census boundaries. In any event, we have applied the most powerful method available and one with confirmed high levels of statistical power (Alexander et al, 1996) to the diverse datasets and failed to find evidence of substantial clustering. The first and very important conclusion is that individual clusters such as those found at Sellafield (COMARE, 1996) and Krümmel (Kaatsch et al, 1996) are rare phenomena and deserve serious attention. The point estimate of extra-Poisson variation for Greece is larger than elsewhere and this has been considered separately in more detail (Petridou et al, in 1997).

The present results may be due to data artefacts but we consider this unlikely. Apart from the statistical significance, the best evidence that they are both genuine and aetiologically meaningful comes from further analyses that we have conducted of *all* small areas in which clusters were deemed to be present. These investigations revealed similar space-time interactions within the clusters (Alexander et al, 1998) to those that had been observed previously in data from the UK, 1966–83 (Alexander, 1992), and cannot readily be explained unless CL has an infectious origin. Further, cluster areas, when compared with control areas, were associated with demographic factors that have been the foundation for the remarkable series of studies by Kinlen and colleagues (Kinlen, 1995; Kinlen et al, 1995). These results are being reported elsewhere (Alexander et al, submitted).

It is possible that the low level of clustering we have observed is attributable to aetiological factors involving only a minority of cases. However, previous papers indicate, at least, that cases influenced by these factors are geographically widespread; for example Kinlen has found an excess of cases in all the situations of population mixing which he has studied. A quantitative ecological analysis of area indices of mobility and leukaemia risk has found the two to be associated in general (Stiller and Boyle, 1996) and not just in extreme instances. If common aetiological pathways generate clustering then the small magnitude is probably the result of one of the other factors noted above, especially migration subsequent to exposure and/or effects restricted in time. Clearly the aetiological exposures do not occur at the date of diagnosis and hence they need not occur while living at the same address. Analyses of complete residential histories should be more powerful for investigating whether children who develop leukaemia have lived close together at some point before their diagnosis. No such analysis has been performed for CL, although one was originally planned for EUROCLUS. Data for Scotland and the South of England are now available and analyses are in progress.

The focus on 'intermediate' areas, although not a prior hypothesis, is consistent with several reports of clusters in 'dormitory suburbs' in the UK (Barclay, 1987; Alexander et al, 1990; Oliver et al, 1992), although these do not appear to have been noted in other countries before this project. This is consistent with a causative infectious agent tending to be endemic in the most densely populated urban areas and unable to generate epidemics in the most rural areas. These post hoc results of exploratory data analysis will require confirmation by independent studies. If confirmed, further study of, for example, community size and population density should provide clues to the transmission and epidemicity parameters of the agent.

The present results fail to confirm our own prior hypotheses that clustering would apply specifically to the childhood peak of ALL that was predicted by biological considerations (Greaves, 1988) and epidemiological studies including some (Kinlen, 1988; Stiller and Boyle, 1996; Alexander et al, 1997) but not all (Kinlen, 1995) of population mixing. The interplay between cases in different subgroups suggest that the same exposures may form part of the aetiological pathway for cases for CL arising at different ages and of distinct cell types, and including, in particular, some infant cases. Further investigation is required.

### ACKNOWLEDGEMENTS

The coordination of this project is funded by the European Union under its BIOMED programme of Concerted Actions as Project Number PL93-1785*26.02.1993. Dr Freda Alexander is also partially supported by the Leukaemia Research Fund and the Kay Kendall Leukaemia Fund. The Australian Paediatric Cancer Registry is supported by the Queensland Cancer Fund. The data for Greece were generated with contributions from Professor S Haida, Professor M Kalmanti, Dr D Koliouskas, Dr H Kosmidi

© Cancer Research Campaign 1998

and Dr F Piperopoulou. The Childhood Cancer Research Group is supported by the UK Departments of Health. The Childhood Cancer Registry of Piedmont is supported by the Italian National Council, contract 95.00449.PF39 and by the Italian Association for Cancer Research. The collaboration of Dr G Couillault and Dr Maynadie with Professor Carli is acknowledged. The Childhood Cancer Registry of Valencia is supported by the Conselleria de Sanitat i Consum of the Generalitat Valenciana. The contribution of Professor Boyle was within the framework of support of the Associazione Italiana per la Ricerca sul Cancro (AIRC). Dr Colin Muirhead is thanked for his contributions to meetings of the Statistics Subcommittee for this project. Mrs Rosemarie Bland and Miss Patricia Bisset are thanked for preparing the manuscript and Mr Darren Downing for assisting with computer graphics.

### REFERENCES

Ahlbom A, Feychting M, Koskenvuo M, Olsen JH, Pukkala E, Schlugen G and Verkasalo P (1993). Electromagnetic fields and childhood cancer. *Lancet* **342**: 1295–1296

Alexander FE (1992) Space-time clustering of childhood acute lymphoblastic leukaemia: indirect evidence for a transmissible agent. *Br J Cancer* **65**: 589–592

Alexander FE (1993) Viruses, clusters and clustering of childhood leukaemia: a new perspective? *Eur J Cancer* **29**: 1424–1443

Alexander FE and Boyle P (1996) *Statistical Methods of Investigating Localised Clustering of Disease*, IARC, Lyon

Alexander FE, Ricketts TJ, McKinney PA and Cartwright RA (1990). Community lifestyle characteristics and risk of acute lymphoblastic leukaemia in children. *Lancet* **336**: 1457–1462

Alexander FE, Wray N, Boyle P, Carli P-M, Coebergh JW, Draper G, Ekbom A, Levi F, McKinney PA, Michaelis J, Petridou E, Peris-Bouet R, Pukkala E, Storm H, Terracini B and Vatten L on behalf of the EUROCLUS project. (1996) Clustering of childhood leukaemia: a European study in progress. *J Epidemiol Biostatist* **1**: 13–24

Alexander FE, Chan LC, Lam TH, Yuen P, Leung NK, Ha SY, Yuen HL, Li CK, Lau YL and Greaves HF (1997) Clustering of childhood leukaemia in Hong Kong: association with the childhood peak and common acute lymphoblastic leukaemia and with population mixing. *Br J Cancer* **75**: 457–463

Alexander FE, Boyle P, Carli P-M, Coebergh JW, Draper GJ, Ekbom A, Levi F, McKinney PA, McWhirten W, Magnani C, Michaelis J, Olsen JH, Peris-Bonet R, Petridou E, Pukkala E and Vatten L on behalf of the EUROCLUS project (1998) Spatial and temporal patterns in childhood leukaemia: further evidence of an infectious origin. *Br J Cancer*

Alexander FE, Boyle P, Carli P-M, Coebergh JW, Draper GJ, Ekbom A, Levi F, McKinney PA, McWhirter W, Michaelis J, Peris-Bonet R, Petridou E, Pompe-Kirn V, Plesko I, Pukkala E, Rahu M, Storm H, Terracini B, Vatten L and Wray N on behalf of the EUROCLUS project. Demographic factors in small areas containing clusters of childhood leukaemia: results of the EUROCLUS study *Eur J Cancer.* (submitted)

Anderson RM and May RM (1991) *Infectious Diseases of Humans: Dynamics and Control.* OUP: Oxford

Barclay R (1987) Childhood leukaemia in Wessex. *Comm Med* **9**: 279–285

Caldwell GG (1990) Twenty-two years of cancer cluster investigations at the centre for disease control. *Am J Epidemiol* **132**: 543–547

Chen R, Mantel N and Klingberg MA (1984) A study of three techniques for time-space clustering in Hodgkin's disease. *Statist Med* **3**: 263

Committee on Medical Aspects of Radiation in the Environment (COMARE) (1996) *Fourth Report.* HMSO: London

Doll R (1989). The epidemiology of childhood leukaemia. *J Royal Statis Soc Series A* **152**: 341–351

Doll R, Evans HJ and Darby SC (1994) Paternal exposure not to blame. *Nature* **367**: 678–680

Draper G (1991) *The Geographical Epidemiology of Childhood Leukaemia and non-Hodgkin Lymphomas in Great Britain, 1966–83.* OPCS: HMSO, London

Esteve J, Benhamou E and Raymond L (1994) *Statistical Methods in Cancer Research Volume IV Descriptive Epidemiology.* IARC Scientific Publication **128**: Lyon

Gardner MJ (1989) Review of reported increases of childhood cancer rates in the vicinity of nuclear installations in the UK. *J Royal Statist Soc* **152**: 307–325

Gardner MJ, Snee MP, Hall AJ, Powell CA, Downes S and Terrell JD (1990) Results of case-control study of leukaemia and lymphoma among young people near Sellafield nuclear plant in West Cumbria. *Br Med J* **300**: 423–439

Greaves MF (1988) Speculations on the cause of childhood acute leukaemia. *Leukaemia* **2**: 120–125

Greaves MF and Alexander FE (1993) An infectious etiology for common acute lymphoblastic leukaemia in childhood? *Leukaemia* **7**: 349–360

Kaatsch P, Kaletsch U, Krummenhauer F, Meineut R, Miesner A, Haaf G and Michaelis J (1996). Case-control study of childhood leukaemia in lower Saxony, Germany. Basic considerations, methodolgy and summary of results. *Klin Paediatr* **208**: 179–185

Kinlen LJ (1988) Evidence for an infective cause of childhood leukaemia: comparison of a Scottish New Town with nuclear reprocessing sites in Britain. *Lancet* **2**: 1323–1327.

Kinlen LJ (1995) Epidemiological evidence for an infective basis in childhood leukaemia. *Br J Cancer* **71**: 1–5

Kinlen LJ, Dickson M and Stiller CA (1995) Childhood leukaemia and non-Hodgkin's lymphoma near large rural construction sites, with a comparison with Sellafield nuclear site. *BMJ* **310**: 763–768

Knox EG (1964) The detection of space-time interactions. *Appl Statist* **13**: 25–29

Lagakos SW, Wessen BJ and Zelen M (1986) An analysis of contaminated well water and health effects in Woborn, Massachusetts. *J Am Statist Assoc* **81**: 583–596

Michaelis J, Keller B, Haaf G and Kaatsch P (1992) Incidence of childhood malignancies in the vicinity of West German nuclear power plants. *Cancer Caus Co* **3**: 255–263.

Muirhead C and Butland BK (1996) The Potthoff-Whittinghill method. In *Statistical Methods of Investigating Localised Clustering of Disease*, Alexander, FE and Boyle P (eds). IARC, Lyon.

Mulder YM, Drijver M and Kreis IA (1994) Case-control study on the association between a cluster of childhood hematopoietic malignancies and local environmental factors in Aalsmeer, The Netherlands. *J Epidemiol Comm Heath* **48**: 161–165

Oliver MA, Muir KR, Webster R, Parkes SE, Cameron AN, Stevens MCG and Mann JR (1992) A geostatistical approach to the analysis of patterns of rare disease. *J Pub Health Med*, **14**: 280–289

Parkin DM, Stiller CA, Draper GJ, Bieber CA, Terracini B and Young JL (1988) *International Incidence of Childhood Cancer.* IARC, Publication No. 87: Lyon

Parkin DM, Clayton D, Black RJ and Masuyer E (1996) Childhood leukaemia in Europe after Chernobyl: 5 year follow-up. *Br J Cancer* **73**: 1006–1012.

Petridou E, Alexander FE, Trichopoulos D, Revinthi K, Dessiprys N, Wray N, Haidas S, Koliouskas D, Kosmidis H, Piperopoulou F and Tzortzatou F (1997) Aggregation of childhood leukaemia in geographical areas of Greece. *Cancer Causes Contrl.* **8**: 239–245

Ross JA, Potter JD and Robinson LL (1994) Infant leukaemia, topoisomerase II inhibitors, and the MLL gene. *J Natl Cancer Inst USA* **86**: 1678–1680

Rothman K (1990) A sobering start for the cluster-busters' conference. *Am J Epidemiol* **132**: s6–s13

Stiller CA and Boyle PJ (1996) Effects of population mixing and socio-economic status in England and Wales, 1979–85 on lymphoblastic leukaemia in children. *Br Med J* **313**: 1297–1300

Temin HM (1992) Keynote Address: Why are there so many leukaemia viruses? *Leukaemia* **6**: 54–55

### APPENDIX 1: COLLABORATORS IN THE EUROCLUS PROJECT

| | |
|---|---|
| Australia | Cancer Registry of Queensland |
| | Dr W McWhirter |
| Denmark | Danish Cancer Registry |
| | Dr H Storm |
| | Dr JH Olsen |
| England and Wales | Childhood Cancer Group |
| | Dr GJ Draper |
| | Dr CA Stiller |
| Estonia | Department of Epidemiology and Biostatistics |
| | Institute of Experimental and Clinical Medicine |
| | Professor M Rahu |

© Cancer Research Campaign 1998

| | |
|---|---|
| Finland | Finnish Cancer Registry<br>Dr E Pukkala<br>Dr L Teppo |
| France | Registry of Haematopoietic Malignancies<br>Professor PM Carli<br>Dr G Couillault<br>Dr M Maynadié |
| Germany | National Register of Childhood Malignancies<br>Professor Dr J Michaelis<br>Dr I Schmidtmann |
| Greece | Special Data Collection<br>Dr E Petridou |
| Italy | European Institute of Oncology<br>Professor P Boyle<br>Childhood Cancer Registry of Piedmont<br>Professor B Terracini<br>Dr C Magnani |
| Netherlands | Dutch Childhood Leukaemia Study Group<br>Dr A Van Der-Does-Van Den Berg<br>Department of Epidemiology and Biostatistics,<br>Erasmus University<br>Dr JW Coebergh |
| Norway | Norwegian Cancer Registry<br>Dr L Vatten |
| Scotland | Co-ordinating Centre<br>Dr F E Alexander<br>Dr N Wray<br>Scottish Cancer Registry<br>Dr D Brewster<br>Dr P McKinney |
| Slovakia | The National Cancer Registry of Slovakia<br>Dr I Plěsko |
| Slovenia | Cancer Registry of Slovenia<br>Prof Dr V Pompe-Kirn |
| Spain | Childhood Tumour Registry of Valencia<br>Dr R Peris-Bonet |
| Sweden | Department of Cancer Epidemiology,<br>University of Uppsala<br>Dr H-O Adami<br>Dr A Ekbom<br>Swedish Cancer Registry<br>Dr J Bring |
| Switzerland | Registres Vaudois et Neuchatelois des Tumeurs<br>Dr F Levi |

© Cancer Research Campaign 1998